# MRI-CT fusion-based digital true hip reconstruction in pediatric developmental dysplasia of the hip: a preliminary feasibility report

**DOI:** 10.3389/fsurg.2026.1952183

**Published:** 2026-09-22

**Authors:** Wei Wang, WenQiang Xu, ChaoYu Liu, YongFei Fan, HaiYang Yu

**Affiliations:** Department of Orthopaedic Surgery, Anhui Provincial Clinical Research Center for Spinal Deformities, Fuyang People’s Hospital, Fuyang People’s Hospital Affiliated to Anhui Medical University, Fuyang, Anhui, China

**Keywords:** 3D printing, developmental dysplasia of the hip, magnetic resonance imaging, osteotomy, pediatric

## Abstract

**Purpose:**

To explore the early clinical feasibility and safety of MRI-CT fusion-based digital “osseous-cartilaginous” hip reconstruction in pediatric developmental dysplasia of the hip (DDH).

**Methods:**

A retrospective analysis was performed on 15 pediatric DDH patients (1 male, 14 females; age 2–12 years, mean 6.06 ± 3.32 years) treated between January 2017 and December 2024, including 12 unilateral and 3 bilateral cases (18 hips total). Preoperative CT and MRI data were fused to construct individualized three-dimensional (3D) “osseous-cartilaginous” hip models, followed by hybrid 3D printing for physical model fabrication. Measurements included femoral neck-shaft angle, anteversion angle, and both osseous and cartilaginous acetabular parameters (acetabular index and coverage rate) to guide personalized osteotomy planning and computer-aided guide plate design. Surgical parameters (operative time, blood loss, fluoroscopy times) and postoperative complications were recorded. Functional outcomes were evaluated using the Mackay scoring system.

**Results:**

Preoperative measurements showed statistically significant differences between osseous and cartilaginous parameters (acetabular index: 30.77° ± 4.98° vs. 19.07° ± 4.03°; coverage rate: 54.67% ± 4.89% vs. 76.78% ± 8.29%; both *P* < 0.05). All 18 hips underwent successful surgery as planned. The mean operative time was 2.64 ± 0.74 h, with an average blood loss of 291.67 ± 184.11 mL and fluoroscopy times of 8.50 ± 2.36. Postoperatively, the neck-shaft angle improved to 132.24° ± 8.26°, and the osseous acetabular index decreased significantly to 15.40° ± 4.01° (*P* < 0.05). All patient-specific guides were successfully seated intraoperatively without adjustment. No major complications occurred during follow-up. Minor complications were recorded in 4 of 18 hips (22.2%), including transient femoral nerve palsy (*n* = 2), superficial wound dehiscence with fat liquefaction (*n* = 1), and grade I pressure sore related to spica cast immobilization (*n* = 1), all of which resolved with conservative management. The Mackay scoring system showed excellent outcomes in 16 hips and good in 2 (excellent-good rate: 100%).

**Conclusion:**

MRI-CT fusion-based digital technology offers a feasible and safe approach for individualized surgical planning in pediatric DDH, with satisfactory short-term clinical outcomes. However, as a preliminary case series without quantitative geometric validation, further prospective studies are warranted.

## Introduction

Developmental dysplasia of the hip (DDH) is managed using a staged approach, including early Pavlik harness immobilization, closed or open reduction, and subsequent periacetabular osteotomy for deformity correction. The primary therapeutic goal is to achieve and maintain stable concentric hip reduction, thereby optimizing the developmental environment of the femoral head and acetabulum while minimizing or preventing long-term osteoarthritis risks (1). Traditional preoperative assessments rely on imaging modalities such as radiography, computed tomography (CT), and magnetic resonance imaging (MRI), which are often limited to two-dimensional representations and thus may fail to comprehensively evaluate the complex three-dimensional (3D) anatomy of the hip (2).

Advancements in digital technology have facilitated its integration into pediatric DDH surgical planning, particularly through the synergistic application of 3D printing and computer-assisted surgical design, which can potentially enhance the precision and success rates of periacetabular osteotomies (3). However, conventional 3D modeling of the pediatric hip, predominantly based on preoperative CT data, reconstructs only osseous structures while neglecting the articular cartilage and epiphyseal plates. This limitation may result in inaccurate representation of true hip pathology and underestimation of the actual acetabular-femoral head contact and coverage areas. Given MRI's superior resolution for cartilage and epiphyseal visualization, the present study utilized preoperative 3D CT and MRI datasets to construct a comprehensive pediatric “osseous-cartilaginous” 3D hip model using Mimics and Geomagic software. This approach enables personalized osteotomy planning and computer-aided guide design, aiming to achieve patient-specific periacetabular osteotomies through an integrated digital and physical workflow. We report our preliminary clinical experience with this technique.

## Patients and methods

### Study design and patients

A retrospective analysis was conducted on the clinical data of 15 pediatric patients (18 hips) with DDH who underwent osteotomy at our institution between January 2017 and December 2024. The cohort comprised 1 male and 14 females, aged 2–12 years (mean: 6.06 ± 3.32 years), including 12 unilateral and 3 bilateral cases. During the same study period, a total of 47 DDH patients underwent surgical treatment at our center; 32 were excluded due to: (1) incomplete MRI data or inability to cooperate with MRI scanning without sedation (*n* = 18); (2) parental refusal of 3D-printing-assisted surgery (*n* = 9); or (3) age < 18 months or neuromuscular comorbidities (*n* = 5).

The inclusion criteria were: (1) meeting the diagnostic criteria for DDH; (2) age ≥ 18 months; and (3) informed consent obtained from guardians. The exclusion criteria were neuromuscular disorders and surgical contraindications. This study was reviewed and approved by the Institutional Ethics Committee of our hospital. All procedures performed were in accordance with the ethical standards of the institutional research committee and with the 1964 Helsinki Declaration and its later amendments.

### Imaging acquisition and 3D model reconstruction

Standard radiographic evaluations included anteroposterior (AP) pelvic radiographs, hip radiographs in abduction-internal rotation and frog-leg positions, and full-length radiographs of both lower extremities. Dual-hip CT scans were performed to acquire raw DICOM data covering the distal femur (parameters: 100 kV, 80–90 mAs; matrix 512 × 512; slice thickness 1 mm; pitch 0.28). MRI examinations were conducted using a Philips 3.0 T superconducting scanner with the protocol cs_3D_VIEW_PDW_Tra (scan parameters: TE 1.93 ms, TR 4.01 ms, duration 15 min, slice thickness 1 mm, FOV 256 mm × 25, T1 star-vibe sequence).

DICOM files from CT and MRI were imported into Mimics software (Materialise, Belgium), where multimodal registration and fusion techniques were applied to spatially align images from both modalities. Bone structures were segmented via threshold-based methods to extract high-density osseous tissues, whereas cartilage regions were delineated using region-growing algorithms. A comprehensive “osseous-cartilaginous” 3D hip model was constructed by integrating these segmented structures (Figure 1). The fusion accuracy was visually confirmed by overlaying the fused model onto original cross-sectional images in three orthogonal planes to ensure anatomical correspondence.

**Figure 1:**
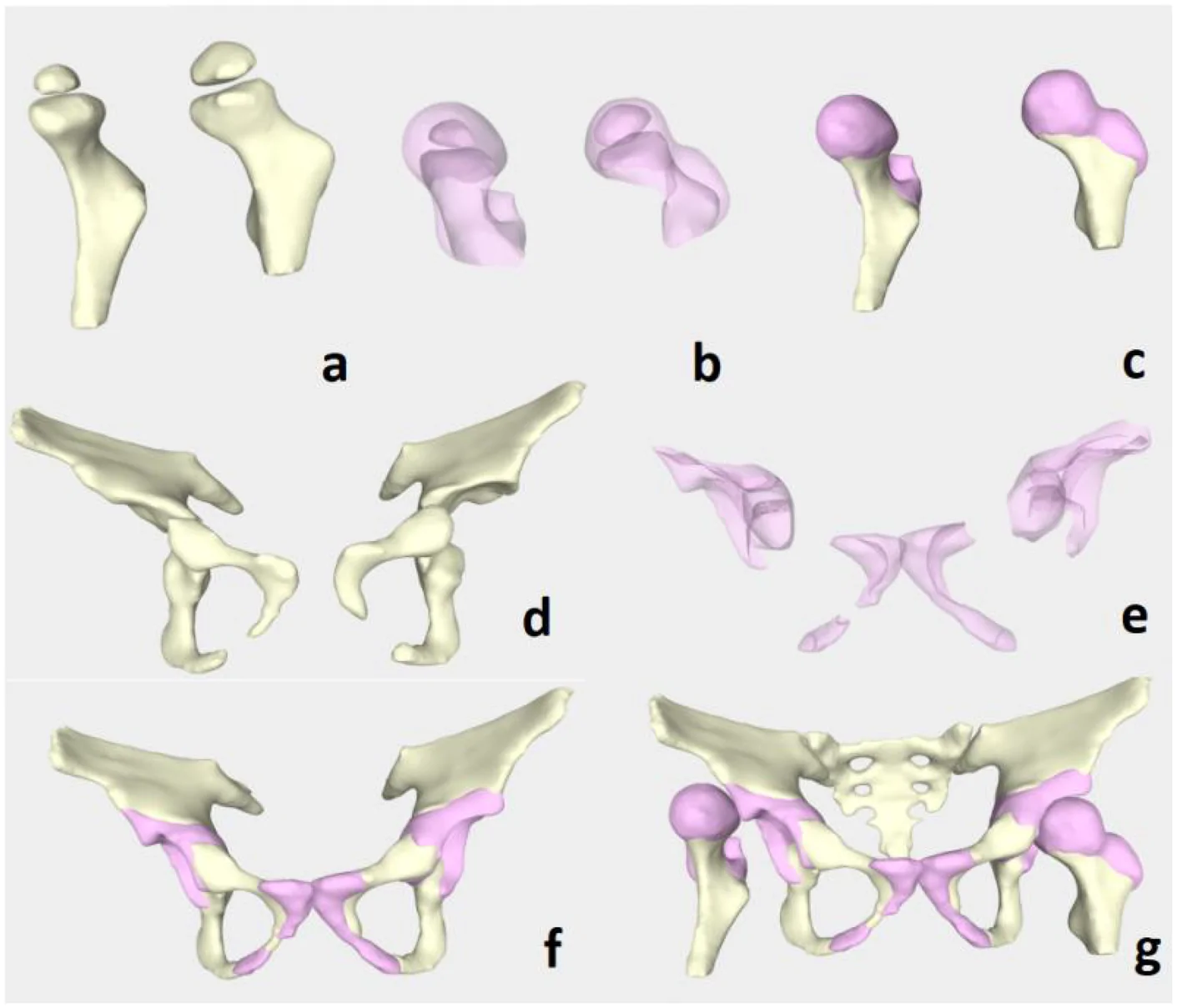
MRI-CT hybrid reconstruction technique: **(a)** CT-based osseous reconstruction of the femoral part; **(b)** MRI-based cartilage reconstruction of the femoral part; **(c)** synthetic matching; **(d)** CT-based osseous reconstruction of the acetabular part; **(e)** MRI-based cartilage reconstruction of the acetabular part; **(f)** synthetic matching; **(g)** secondary synthesis to construct a 3D “osseous-cartilaginous” model of the hip joint.

### Surgical planning and guide plate design

Based on the fused 3D model, we obtained the neck-shaft angle, anteversion angle, and both osseous and cartilaginous indicators (acetabular index and acetabular coverage rate). Different surgical plans (Salter, Steel triple, or Pemberton osteotomy, with or without proximal femoral osteotomy) were simulated on the model to determine the optimal approach (Figure 2).

**Figure 2:**
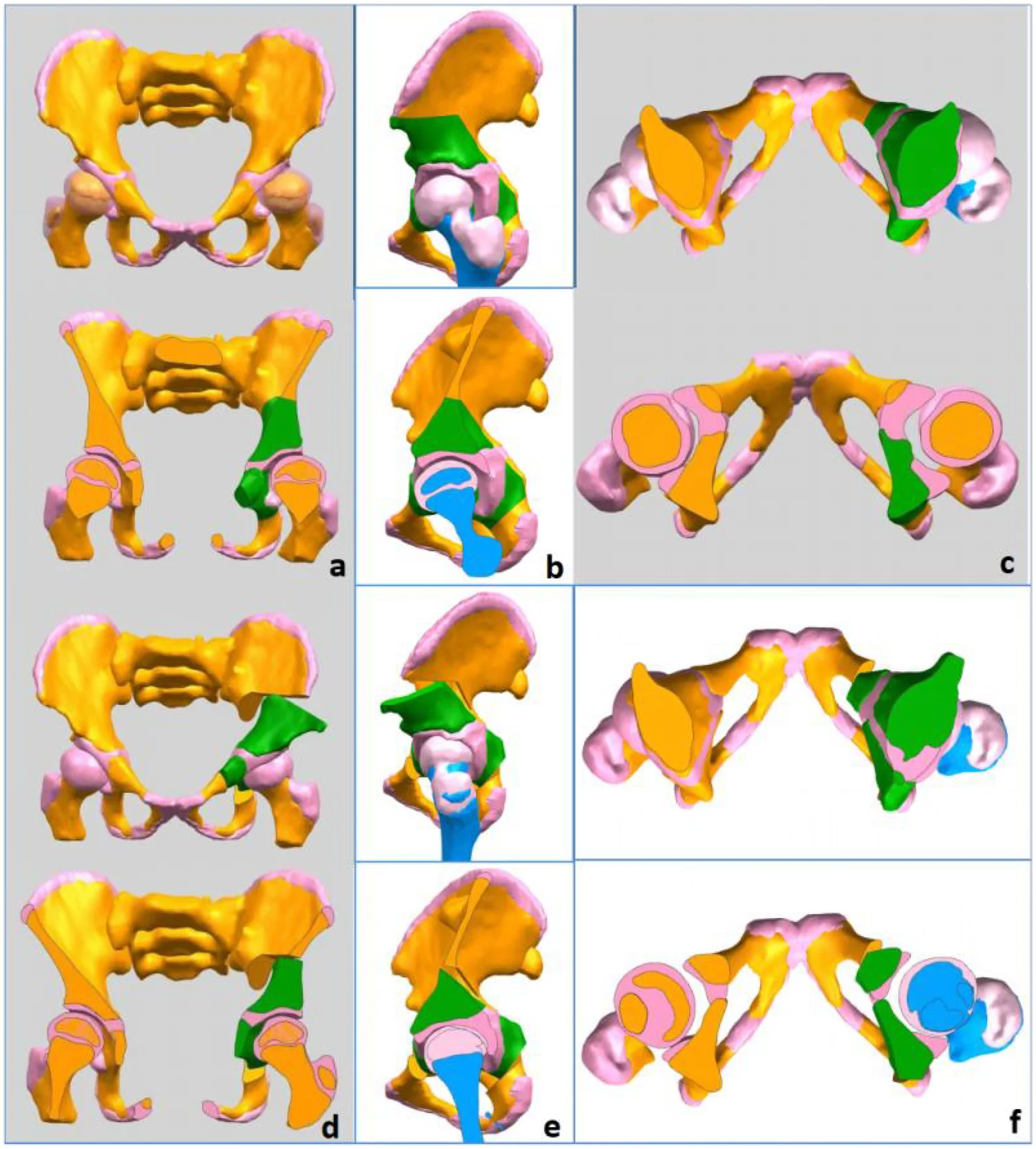
**(a–c)** three-plane views before simulated osteotomy; **(d–f)** three-plane views after simulated osteotomy.

For guide plate design, a standardized workflow was adopted: (1) the osteotomy line and rotation/translation vectors were determined on the virtual model; (2) a personalized guide plate was designed to fit specific bony landmarks (e.g., iliac crest, ischial ramus, and anterior inferior iliac spine) to ensure stable intraoperative seating; (3) guide plates were designed with reserved Kirschner-wire (K-wire) temporary fixation holes to maintain reduction after osteotomy; (4) the plates were printed using medical-grade resin, packaged, and sterilized for intraoperative use. To measure femoral neck anteversion, the contralateral normal side (if unaffected) was used as the reference. Based on this, a simulated proximal femoral osteotomy was performed to reconstruct anteversion and neck-shaft angle, and an appropriate plate was selected for fixation.

### Surgical procedure and postoperative management

All surgeries were performed by the same senior surgical team. After exposure, the pre-sterilized 3D-printed guide plate was positioned onto the corresponding anatomical landmarks. Intraoperative success was defined as stable seating of the guide plate without gross mobility and accurate placement of K-wires through the pre-designed holes.

After osteotomy and reduction, a hip spica cast or orthosis was used for fixation. The external fixation device was removed 6–8 weeks later, and functional joint exercises were initiated. Two to three months later, weight-bearing exercises were started gradually. Regular x-ray examinations were performed. Once the osteotomy site had fully healed, internal fixation devices were removed.

### Outcome measures and statistical analysis

All relevant postoperative complications (including infection, nerve injury, redislocation, implant failure, and nonunion) were meticulously recorded. Functional outcomes at the last follow-up were evaluated using the Mackay scoring system.

Statistical analysis was performed using SPSS version 27.0 (IBM Corp., Armonk, NY, USA). Quantitative indicators are presented as mean ± standard deviation (SD). Paired-sample *t*-tests were used to compare preoperative and postoperative parameters, as well as osseous versus cartilaginous indicators. A *P*-value < 0.05 was considered statistically significant.

## Results

### Preoperative imaging parameters

Preoperative measurements revealed statistically significant differences between the osseous acetabular index (OAI) and cartilaginous acetabular index (CAI) (*P* < 0.05), as well as between the osseous acetabular coverage (OAC) and cartilaginous acetabular coverage (CAC) (*P* < 0.05) (Table 1). These findings indicate that the cartilage around the acetabulum provides better containment of the femoral head than suggested by bone measurements alone.

**Table 1:** Comparison of osseous versus cartilaginous acetabular parameters (mean ± SD).

Parameter	Osseous	Cartilaginous	*t*-value	*P*-value
Acetabular Index (°)	30.77 ± 4.98	19.07 ± 4.03	8.459	<0.001
Acetabular Coverage (%)	54.67 ± 4.89	76.78 ± 8.29	−16.466	<0.001

### Surgical outcomes and guide plate performance

All 15 pediatric patients (18 hips) underwent successful surgery. Among the 18 hips, 6 underwent Salter osteotomy (4 combined with proximal femoral osteotomy), 10 underwent Steel triple osteotomy (4 combined with femoral osteotomy), and 2 underwent Pemberton osteotomy (1 combined with femoral osteotomy). All 18 patient-specific guide plates were successfully seated and applied intraoperatively without any need for intraoperative adjustment or abandonment due to soft tissue interference or mismatch with the actual anatomy.

The mean operative time, blood loss, intraoperative fluoroscopy times, and follow-up duration are shown in Table 2. The postoperative OAI was 15.40° ± 4.01°, which was significantly improved compared with the preoperative value (*t* = 11.290, *P* < 0.001).

**Table 2:** Preoperative, intraoperative, and postoperative data of patients (Mean ± SD).

Variable	Preoperative	Intraoperative	Postoperative
Age (year)	6.06 ± 3.32		
Sex			
Boy	1		
Girl	14		
Affected side			
Left	10		
Right	8		
Surgical method			
Salter	2		
Salter + proximal femoral osteotomy	4		
Steel	6		
Steel + proximal femoral osteotomy	4		
Pemberton	1		
Pemberton + proximal femoral osteotomy	1		
Fluoroscopy times		8.50 ± 2.36	
Operative time (4)		2.64 ± 0.74	
Blood loss (mL)		291.67 ± 184.11	
Follow-up (month)			41.78 ± 14.7
Mackay scoring system			
Excellent			16
Good			2
Fair			0
Poor			0

### Complications

No major complications, such as deep surgical site infection, neurovascular injury, redislocation, implant failure, or nonunion, were observed during the perioperative period or throughout the follow-up period. However, minor complications were recorded in 4 of 18 hips (22.2%). Specifically, transient femoral nerve palsy (quadriceps strength grade IV, resolving spontaneously within 6 weeks without sequelae) occurred in 2 patients, which was attributed to intraoperative retraction during pelvic osteotomy. Superficial wound dehiscence with fat liquefaction occurred in 1 patient, which healed uneventfully after enhanced wound care without deep infection. Grade I pressure sore related to spica cast immobilization occurred in 1 patient, which resolved completely after local wound care. All minor complications were successfully managed with conservative measures, and none required surgical intervention or prolonged hospitalization. No patient developed avascular necrosis of the femoral head during the follow-up period.

### Functional outcomes

According to the Mackay scoring system, 16 hips were rated as excellent and 2 as good, resulting in an excellent-good rate of 100% at the final follow-up. Representative preoperative and postoperative radiographic outcomes are illustrated in Figures 3, 4.

**Figure 3:**
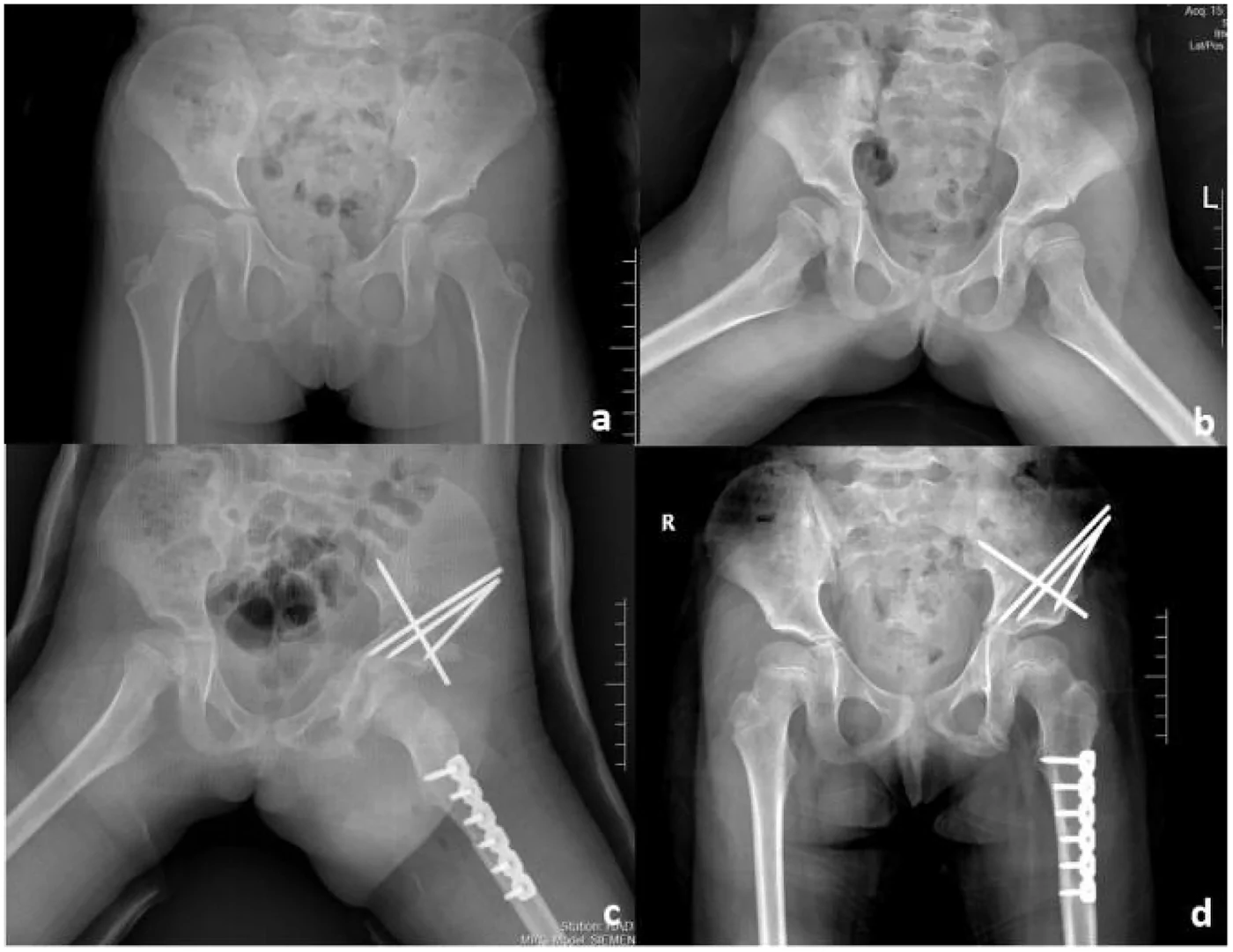
A 5-year-old girl: **(a)** AP radiograph of the hip joint before surgery; **(b)** frog-leg position; **(c)** AP radiograph after surgery; **(d)** healing condition of the osteotomy site after surgery.

**Figure 4:**
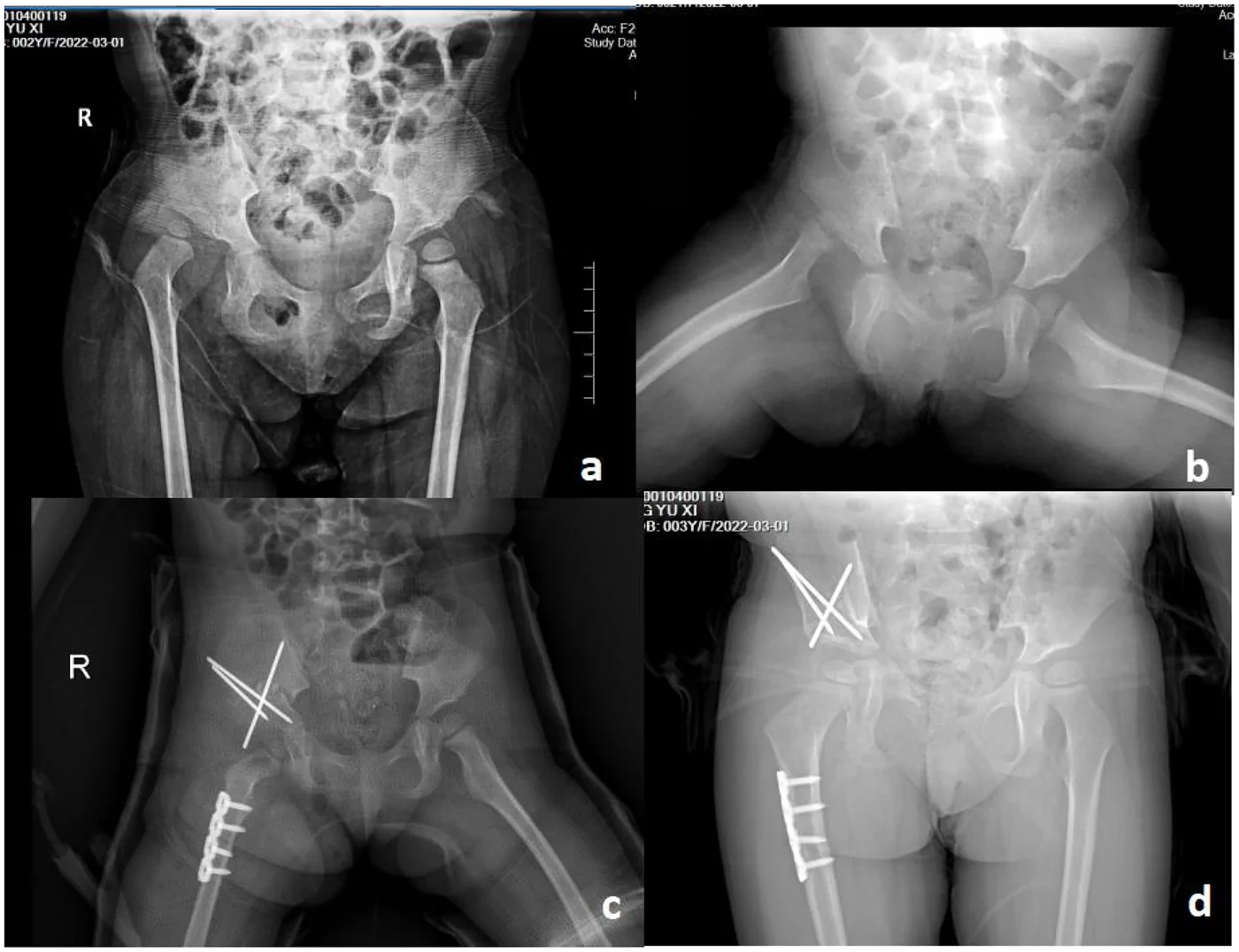
A 2-year-old girl: **(a)** AP radiograph of the hip joint before surgery; **(b)** frog-leg position; **(c)** AP radiograph after surgery; **(d)** healing condition of the osteotomy site after surgery.

## Discussion

The selection of osteotomy technique for DDH involves multiple considerations. Beyond patient age and surgeon familiarity, a critical objective factor lies in preoperative radiographic observation and measurement of the pediatric hip joint. Accurate anatomical data are the foundation for precise osteotomy, which ensures favorable clinical outcomes. Consequently, obtaining intuitive and reliable images that reflect the true morphology of the hip joint is paramount. Early imaging techniques, including anteroposterior, abduction-internal rotation, frog-leg, and faux-profile radiographs, have evolved to incorporate advanced three-dimensional CT and MRI, all aimed at capturing the hip's anatomical structure and dynamic changes with greater fidelity. Following thorough data analysis and surgical planning, another clinical challenge is to effectively predict osteotomy outcomes (4).

With the continuous advancement of computer hardware and software technologies, medical imaging has achieved significant breakthroughs. Currently, research focus in the medical field is shifting toward the integration of engineering concepts into clinical practice, with digital modeling and 3D printing technologies representing a paradigm of such interdisciplinary convergence (5). As 3D printing technology has been extensively applied in the orthopedic field, numerous scholars have also adopted this technology for personalized diagnosis and treatment of pediatric DDH. The summarized benefits include: (1) gaining a deeper understanding of complex anatomical structures (5, 6), facilitating communication with patients and families regarding the condition and treatment plan (7); (2) validating and optimizing different surgical techniques on life-sized printed models in a stress-free environment (8); (3) conducting detailed preoperative planning and simulated osteotomy to ensure more accurate dimensions of bone grafts, facilitate positioning, and pre-select internal implants (9); and (4) fabricating and applying osteotomy guide plates (10). Multiple reports have indicated that compared with conventional treatment groups, the application of 3D printing technology has led to reductions in surgical time, blood loss, and radiation exposure (11–13).

However, CT-based 3D printing has inherent limitations in pediatric hip surgery. Since CT data can only reconstruct osseous structures, while the acetabulum and femur in children contain substantial cartilage and epiphyses—with the proportion being higher in younger children—the cartilage components are automatically excluded during modeling and printing. This may result in an inability to truly reproduce the three-dimensional deformity, raising doubts about its accuracy (14). In contrast, MRI is not restricted by the degree of ossification and possesses multiplanar imaging capabilities (15). It offers good resolution of the cartilage and epiphyses of the acetabulum and proximal femur. Given MRI's excellent tissue differentiation and lack of ionizing radiation, it holds great potential in 3D printing applications.

In previous studies, MRI-based data integration, modeling, and printing were mostly applied to soft tissue diseases, such as cardiac and prostate tumors. In the pediatric hip field, several scholars have conducted series of studies on MRI-guided three-dimensional reconstruction and printing. Sukagoshi et al. (14) used 3D MRI to evaluate femoral head cartilage morphology in DDH patients after Pavlik harness failure, finding that the dislocated femoral head was smaller and more irregular than the healthy side. Another MRI-based 3D study suggested that predicting future acetabular growth based on cartilage shape was more reliable than bone-based predictions from x-rays (16). Some scholars have compared MRI-based 3D printed models with CT-based models and found that average surface differences for both the acetabulum and femur were less than 0.5 mm; importantly, MRI-based models showed better correlation for anterior, posterior, and total femoral head coverage, as well as anteversion angle (17). MRI-based 3D printed models have also been successfully applied in preoperative planning for slipped capital femoral epiphysis, avoiding radiation exposure and significantly reducing surgical time and blood loss with satisfactory clinical outcomes (18).

Given the cartilage-rich nature of the pediatric acetabulum and proximal femur, we employed MRI for detailed cartilage information. Additionally, because cortical bone has an extremely short T2 relaxation time, resulting in limited MRI signal, we also acquired CT data for fully ossified areas (19). The DICOM data from both modalities were imported into Mimics software. After threshold segmentation, dynamic region growing, and Boolean operations, the processed data were exported in STL format and imported into Geomagic software. After unifying the coordinate system, registration was performed to construct a 3D “osseous-cartilaginous” model. This model utilizes MRI to capture cartilage details and CT to present osseous structures, achieving complementary data acquisition. It can more accurately reflect the 3D deformity and pathological state of the hip joint, allowing observations from any angle, adjustable transparency, and pseudo-color annotations.

Femoral head coverage rate is a crucial parameter in treating DDH patients (20). The primary objective of corrective surgery is to increase femoral head coverage and optimize the orientation of the weight-bearing area, thereby reducing joint contact stress and lowering the risk of early-onset osteoarthritis (21, 22). Previously, x-rays and CT scans were commonly used to calculate femoral head coverage, which only considered osseous coverage. The measurement of the cartilage interface obtained through MRI scans is a single-plane assessment and lacks three-dimensionality. Owing to these limitations, the accuracy of the acetabular coverage rate is questionable after downward compression and rotation of the osteotomy end. The “osseous-cartilaginous” 3D model can clearly present the overall morphology and spatial relationships of the hip joint, enabling an accurate assessment of true femoral head coverage and potential defect areas. Based on this model, it is possible to simulate the impact of different osteotomy angles (downward compression, rotation, and lateral displacement) on femoral head coverage. If necessary, proximal femoral osteotomy can be performed in conjunction to enhance postoperative stability, thereby providing a direct basis for surgical planning and reducing trial-and-error costs. For example, in Salter pelvic osteotomy, the model can predict the relationship between the rotation angle of the osteotomy end and changes in the acetabular index, preventing excessive rotation that could lead to excessive gaps or pubic symphysis separation.

### Limitations

We acknowledge several important limitations in this study. First, this is a retrospective case series without a control group (e.g., CT-only or conventional x-ray planning), corresponding to Level IV evidence. Therefore, our results should be interpreted as a preliminary feasibility and safety report rather than proof of superiority over conventional techniques. Second, the sample size is small (15 patients, 18 hips), and there is a significant gender imbalance (1:14 male-to-female ratio). Although this reflects the well-known female predominance in DDH epidemiology, it limits the generalizability of our findings to male patients. Third, and most importantly, we did not perform a systematic quantitative geometric accuracy validation of the fused models, such as surface deviation analysis (e.g., Hausdorff distance) or inter-observer reproducibility (intraclass correlation coefficient) calculations. While we visually confirmed the registration accuracy on orthogonal cross-sections and, more importantly, all 18 patient-specific guides were successfully seated intraoperatively without adjustment—indirectly supporting the clinical adequacy of model fidelity—we recognize that the lack of strict quantitative 3D deviation metrics is a substantive limitation. Future prospective studies should incorporate standardized geometric validation using software-based 3D comparison tools (e.g., Geomagic Control X) to provide higher-level evidence. Fourth, the data processing and segmentation of MRI take a relatively long time and incur additional costs, and specialized training is required for relevant software operation. Additionally, the use of 3D-printed templates may require a larger surgical field for positioning, potentially leading to increased soft tissue dissection and wound-related issues in some patients. Fifth, the accuracy and reliability of the fused 3D models are inherently dependent on the quality of the source imaging data. Factors such as MRI motion artifacts (particularly in young children who may require sedation), magnetic field distortions caused by metal objects or air-tissue interfaces, and boundary inhomogeneity in cartilage segmentation can all potentially affect the fidelity of the reconstructed models (23). Although we implemented visual confirmation of registration accuracy on orthogonal cross-sections and utilized standardized semi-automated segmentation protocols to minimize operator-dependent variability, we acknowledge that inter-observer differences in manual segmentation and variability in imaging quality across patients remain potential sources of error. Future studies should incorporate automated or deep learning-based segmentation techniques to reduce these risks and improve reproducibility (17).

Despite these limitations, MRI-CT composite digital technology for reconstructing the “osseous-cartilaginous” 3D model integrates 3D anatomical information, optimizes surgical planning, enhances execution accuracy, and improves doctor-patient communication. The technical advantages of this method include not only the precision of intraoperative operations but also the entire process of preoperative assessment, intraoperative decision-making, and postoperative management. This offers a more optimized treatment plan for children with DDH, ensuring the effectiveness of their treatment.

## Data Availability

The original contributions presented in the study are included in the article/Supplementary Material, further inquiries can be directed to the corresponding author.
